# Evaluation of the “Resident as teacher” curriculum: a needs assessment in medical education at a large academic institution

**DOI:** 10.12688/mep.20786.1

**Published:** 2025-03-25

**Authors:** Yi Zhen Jia, Véronique Castonguay, Carole Lambert, Jean-Michel Leduc

**Affiliations:** 1 Faculty of Medicine, University of Montreal, Montreal, Québec, Canada

**Keywords:** Resident as teacher, Program evaluation, Needs assessment

## Abstract

**Introduction:**

Strong skills in teaching for residents contribute to increased satisfaction and improved student interest. Few opportunities in medical education are offered at the junior resident level. We aim to evaluate the needs of residents in teaching at Université de Montréal

**Methods:**

A 19 question survey created after a literature review was sent to all 769 current PGY1 to PGY3 residents at UdeM to assess their interest and needs in various aspects of clinical teaching. Descriptive statistics were analyzed to make recommendations for improvements in medical education training.

**Results:**

We received 65 completed surveys (8.5% response rate), mostly in family and internal medicine. Eighty percent of participants were interested in further training in teaching and 58% were interested in a medical education elective. Main skills to be improved are indirect supervision and adapting feedback to different learners. Lack of time was considered by most responders (89%) as the main factor limiting participation in further training. Narrative comments noted the lack of information on medical education resources and lack of recognition by faculty compared to clinical performance or research, particularly in family medicine.

**Conclusion:**

Protected time for varied medical education activities is needed, including better offers for an elective rotation. Information on currently available resources should be more widely circulated. Promoting and recognizing teaching and reserving time for direct supervision by faculty of teaching by junior residents should be encouraged.

## Introduction

Up to half of medical students’ clinical teaching comes from resident physicians, who spend as much as a quarter of their time in this role ^1, 2^. Teaching is a significant part of CanMeds’ core competency of scholar. The importance of proficiency in this area has been demonstrated to contribute to residents’ satisfaction and medical students` interest ^3^. Yet, postgraduate programs have little dedicated time for program-specific “Resident as teacher” curriculum, even though individualized training has been shown to improve resident confidence and performance in their teaching skills and their own learning ^4– 7^. As of date, no formal national standard for this type of curriculum has been developed in Canada.

Current residency academic curriculum is filled with lectures, simulations, procedural skills, and workshops, leaving limited time to add dedicated teaching workshops. Residents are often overwhelmed by competing responsibilities in patient care, research, and administration without forgetting that they are learners themselves. The settings they work in also vary greatly depending on the site and specialty, from the Emergency Department to the OR, each needing specific pedagogical skills. However, it has been established that as little as half a day can have a meaningful impact on resident comfort in teaching ^8^. Regardless of the specialty, a well-built course can help improve the teaching skills of all residents ^4– 7^.

Currently, Université de Montréal (UdeM) offers a mandatory four-hour online training course for all PGY1 residents, with a pre-activity lecture and a post-activity reflective essay. The course is given in a small-group format with a focus on four themes: the role of the resident as a teacher, supervision, helping learners in situations of intimidation, and providing feedback. Although a dedicated one-month rotation in medical education is available for a small subgroup of senior residents, to our knowledge, no other formal pedagogy course is available for junior residents who desire further advancement in clinical teaching.

## Project goal

The goal of this project was to assess the interest of junior residents in medical education and the need to develop further training at our institution. Secondary goals were to evaluate obstacles to participation in such training and satisfaction with the current course. The data gathered will be used to provide broader recommendations for the implementation of medical education curriculum in residency and as a bridge to improve the current curriculum at UdeM. We believe that the recommendations devised from this evaluation will allow crucial changes, translating into greater resident comfort and ability in education, along with increased learner interest. We hope that these recommendations will also be useful for other institutions.

## Methods

An anonymous online survey was developed using data based on a review of current recommendations and standards for “Resident as Teacher” curriculums and surveys in medical education through PubMed ^9– 11^. It included Likert scales, multiple-choice, and narrative questions for a total of 19 questions. The main themes asked were current exposure to medical education, interest in education and self-evaluation of current teaching skills ^12^. Results from a past internal survey of senior residents at UdeM detailing their experience with the currently offered medical education elective were also reviewed to detail questions concerning areas of improvement for the current curriculum. Confidence in teaching skills evaluated in the elective course at UdeM was assessed by using a 5-point Likert scale, where 1 was least comfortable and 5 was most comfortable.

The survey was reviewed independently by two faculty members with strong expertise in medical education and resident training (VC and CL) and tested by three current residents for feedback and improvement in grammar and question precision. The survey was sent by email using the Qualtrics software to all PGY1, PGY2 and PGY3 residents (769 residents) at UdeM. A one-month period was granted for the completion of the survey with weekly reminders, totaling four emails. For data analysis, descriptive statistics were computed for every question, and narrative comments were summarized to extract the main themes. Recommendations were produced to help implement further medical education curricula for residency programs.

## Results

Sixty-five completed surveys (8.5 % response rate) were collected during the allotted time period for a total of 34 PGY1, 20 PGY2, and 11 PGY3 responders. Thirty residents identified as family medicine residents (46 %), 17 as internal medicine (26 %), and 18 were from other specialties (28 %). Thirty-eight percent of respondents had formal training in education beyond the mandatory course offered, mainly through seminars and conferences offered during medical training. Thirty-one percent of residents spent more than two hours teaching every week, mostly in the setting of wards or clinics (71 %).

Eighty percent of residents were rather interested or very interested in further opportunities in training in medical education, particularly through simulations of difficult teaching scenarios (42 %). Supervision of their teaching (18 %), classes on education theory (14 %), online modules (12 %), and small group activities (11 %) also had interest. Fifty-eight percent would be very or extremely interested in a medical education elective. Most residents enjoy teaching (89 %) and agree it plays a crucial role during residency (89 %).

However, the lack of recognition of teaching as a core skill was mentioned in narrative comments, and only 49 % of residents believed it was strongly recognized by faculty. Limited time was a recurring factor in hesitation for more time commitment (89 %), with 71 % of residents interested in participating less than 10 hours/year. Limited knowledge of resources available in medical education was described by responders (0 known journals/resources in 71 % responders).

Residents’ comfort levels for certain tasks were evaluated in this study. Most residents are somewhat comfortable with most skills, especially as professional role models. However, few were very comfortable with all tasks. Notably, helping a learner in difficulty seemed to be an area with which more residents felt less comfortable ( Table 1).

**Table 1.** Level of comfort with teaching skills (N = 65).

	Level of comfort (% of responders)					
	1 (Not at all)	2	3	4	5 (Very)	Not answered
Help a learner in difficulty	5	15	28	40	12	0
Teach technical skills	6	8	37	34	15	0
Offer constructive feedback to learners	0	8	32	48	8	5
Be a professional role model	0	5	23	48	23	2
Evaluate clinical reasoning	0	9	32	48	9	2
Direct supervision of clinical cases	0	5	20	59	15	2
Indirect supervision of clinical cases	2	9	29	49	9	2

Several themes emerged from the narrative analysis.

### Obstacles to training

A lack of time was brought up several times by the respondents. According to the comments, even though interest is strong, making additional courses obligatory would be counterproductive. The lack of recognition by faculty is also a barrier with one resident in family medicine stating that she had to “take vacations to participate in medical student teaching.” Few would be open to facultative sessions in medical education if it counted towards the maximum allowed days off during a rotation, as it would compete with studying, research, and vacations. There is a perceived lack of opportunities at UdeM. Many residents are not entirely comfortable with teaching; yet they do not know where to obtain additional information and training. One responder commented: “I do not know it at all. Maybe publicize more to residents’’. 

### Appreciation of current curriculum

Strong appreciation for the current course was noted among current residents. The early timing in residency training and the small group format were acknowledged positively, as it allowed residents to practice certain scenarios and receive feedback before applying it to medical students in the clinical context. Many found supervisors to be very implicated and open to adapting the curriculum to the residents’ questions. One responder noted that programs should “capitalize on the very good training in PGY1”.

### Skills to improve

Providing personalized feedback adapted to the learner was the skill most sought by responders. One responder believed that it can be difficult to: “Put aside personality differences and really focus on the learner.” Another subject for which additional training would be beneficial was indirect supervision. “It is difficult to redo the history, let the student speak uninterrupted and not forget questions/plan that need to be done” was noted by another responder.

## Discussion

This project represents the first evaluation of current residency training in medical education at UdeM. Great interest in teaching was shown, even at the junior level. Previous studies have shown that participation in workshops increases interest significantly ^4, 6, 8^. Appreciation is present in the current curriculum and should be maintained. There is enthusiasm for all types of teaching activities, as the skills needed varied among residents. Most residents are somewhat comfortable teaching, thanks to the current curriculum and clinical experience. However, few were comfortable with all teaching tasks, with the main ones where additional training would be warranted are offering adapted feedback and indirect supervision according to narrative comments. This suggests a need for varied and flexible courses. Residents spent less time teaching than expected ^1, 2^ probably owing to the junior level of responding residents or different interpretations of the actual time spent. 

Multiple comments by responders brought up a lack of recognition by faculty compared to clinical performance or research ^2, 7, 13^, particularly in family medicine. There are clear differences between family medicine programs and teaching opportunities depending on the influx of students, and it can be difficult to find opportunities. Our findings showed that lack of time was a unifying factor in hesitancy in more mandatory teaching in medical education, consistent with our hypothesis and the current literature ^2, 3^. Most responders would be open to more training if protected time was given or if an elective rotation was offered. There is a similar lack of knowledge regarding the available resources. Even with enthusiasm for teaching, this interest can fade if no opportunities are offered by programs or by the university.

## Limitations and recommendations

The results were comparable to those of the current research from other institutions and countries ^2, 3, 5, 6^. Due to limited response rates, selection bias is possible, with responders more likely to have an interest in medical education. Although we received responses from residents in a variety of specialties, we did not receive any response from some smaller specialties, and our results may not apply to them. This study was conducted at a single institution, in a distinct medical education environment. This may not apply to other institutions with different curricula and resources. Further studies are needed to evaluate whether these results can be replicated. However, based on our data, some general recommendations can be formulated that we believe can be applicable to other settings.

1. Allow flexible and protected time for participation in medical education activities.
2. Build an elective rotation for junior residents who wish to undertake a medical education elective.
3. Offer a variety of activities on different skills in medical education, including, but not limited to, simulations of difficult situations, classes or online modules on teaching theory, and small group activities.
4. Widely circulate information on currently available resources in medical education (journals, supervisors, conferences, and elective courses).
5. Encourage promotion and recognition of teaching in the clinical setting and offer reserved time by faculty for supervision/observation of teaching by junior residents.

## Conclusion

Teaching is a crucial part of residents’ roles in their training. Feedback for the current course was positive. There is strong interest among residents to further their experience with a variety of opportunities and skills in medical education. However, lack of time, opportunities, and access to information were the most significant limiting factors. Recognition of the meaningful role of residents as teachers needs to be encouraged. We plan to use our results and recommendations to help improve the Université de Montreal curriculum of “Residents as Teachers”. We encourage other faculties to evaluate their curricula among their residents and adapt our recommendations to their environment to offer continuous improvement.

## Ethics and consent

### Ethics

This project is considered a program evaluation and was waived from the ethical review by letter on December 19 ^th^, 2023, by the Université de Montréal Ethic committee on research in education and psychology (CEREP).

### Consent

Written informed consent for the study was obtained in the survey. Respondents were informed that results of the survey would be used for the present study.

## Data Availability

Open Science Framework: Survey and data for Evaluation of the resident as teacher curriculum: A needs assessment in medical education at a large academic institution DOI: 10.17605/OSF.IO/2M3NY The project contains the following underlying data: Data MedEd English (Survey answers in Excel format) Survey research project (Survey in Word format) Data are available under the terms of the Creative Commons Zero “No rights reserved” data waiver (CC0 1.0 Public domain dedication)
