# Supplementary material for: Evaluation of the “Resident as teacher” curriculum: a needs assessment in medical education at a large academic institution
Source: MedEdPublish (2016). 2025 Oct 2;15:10. Originally published 2025 Mar 25. [Version 2] doi: 10.12688/mep.20786.2 (PMC12553972; doi:10.12688/mep.20786.2)
Supplement: Peer review history

# Reviewer report: Reviewer response for version 2

**DOI:** 10.21956/mep.22854.r43787

**Published:** 2025-11-14

**Authors:** Sacha Sharp

**Affiliations:** 1 Indiana University School of Medicine, Indianapolis, Indiana, USA

I approve the new version of this manuscript. 

 I do appreciate the authors for their response to a few of the comments previously made. Unfortunately, there is still room in the discussion and recommendations sections for more synthesis of data. However, the article is now clearer and more concise.

Have any limitations of the research been acknowledged?

Yes

Is the study design appropriate and does the work have academic merit?

Partly

Is the work clearly and accurately presented and does it cite the current literature?

Yes

If applicable, is the statistical analysis and its interpretation appropriate?

I cannot comment. A qualified statistician is required.

Are all the source data underlying the results available to ensure full reproducibility?

Yes

Are the conclusions drawn adequately supported by the results?

No

Are sufficient details of methods and analysis provided to allow replication by others?

No

Reviewer Expertise:

NA

I confirm that I have read this submission and believe that I have an appropriate level of expertise to confirm that it is of an acceptable scientific standard.

# Reviewer report: Reviewer response for version 2

**DOI:** 10.21956/mep.22854.r43786

**Published:** 2025-10-28

**Authors:** Nancy Dalgarno

**Affiliations:** 1 Office of Professional Development and Educational Scholarship, Queen's University, Kingston, Ontario, Canada

The changes have certainly improved the manuscript - well done. The only remaining comment I have is in relation to intercoder reliability. You state, "This analysis was done by the primary author (YJ) and the supervising author (JML) and there were minimal inter-researcher differences." Please describe the process used when differences were encountered. For example, did you discuss the conflicts until consensus was reached? Once this has been added the paper, it is, in my opinion ready to be accepted.

Have any limitations of the research been acknowledged?

Yes

Is the study design appropriate and does the work have academic merit?

Yes

Is the work clearly and accurately presented and does it cite the current literature?

Partly

If applicable, is the statistical analysis and its interpretation appropriate?

Partly

Are all the source data underlying the results available to ensure full reproducibility?

No

Are the conclusions drawn adequately supported by the results?

Yes

Are sufficient details of methods and analysis provided to allow replication by others?

Yes

Reviewer Expertise:

Medical education, program evaluation

I confirm that I have read this submission and believe that I have an appropriate level of expertise to confirm that it is of an acceptable scientific standard.

# Reviewer report: Reviewer response for version 2

**DOI:** 10.21956/mep.22854.r43951

**Published:** 2025-10-25

**Authors:** Emre Emekli

**Affiliations:** 1 Eskişehir Osmangazi University, Eskişehir Province, Eskişehir Province, Turkey

The article is generally well-structured, conducted with a clear objective and a logical methodology. This study, which focuses on the evaluation of the “Resident as Teacher” (RaT) program, fills an important gap, particularly within the Canadian context. The research question is well-defined, the methodology section is detailed, and the results are clearly presented. The findings effectively highlight both the strengths of the current curriculum and the areas that need improvement. The feasibility of the proposed solutions enhances the study’s potential contribution to medical education. Overall, the paper demonstrates academic rigor, offers practical recommendations, and is well-written and suitable for indexing.

 Method:

 Themes were identified through “narrative comments,” but the analytical approach (e.g., content analysis, thematic analysis, coding process) is not clearly described. The authors mention strong agreement between the two researchers, but it would be helpful to explain how disagreements were resolved. Clarifying how inter-rater reliability was assessed (e.g., using Cohen’s kappa) would strengthen the study’s credibility.

 Representative quotes are provided; however, including more illustrative examples for each theme would improve the qualitative depth of the analysis.

 Discussion:

 The discussion section is generally well-written, but the connections to the literature are sometimes superficial. The comparison with other Canadian examples (e.g., UBC, McMaster) could be addressed in a more systematic and analytical manner.

Have any limitations of the research been acknowledged?

Yes

Is the study design appropriate and does the work have academic merit?

Yes

Is the work clearly and accurately presented and does it cite the current literature?

Yes

If applicable, is the statistical analysis and its interpretation appropriate?

Yes

Are all the source data underlying the results available to ensure full reproducibility?

Yes

Are the conclusions drawn adequately supported by the results?

Yes

Are sufficient details of methods and analysis provided to allow replication by others?

Partly

Reviewer Expertise:

My primary research areas include medical education, radiology, and the integration of artificial intelligence (AI) and measurement tools into health professions education. I focus particularly on assessment and item generation in medical education, clinical reasoning, and AI-assisted learning and evaluation methods.

I confirm that I have read this submission and believe that I have an appropriate level of expertise to confirm that it is of an acceptable scientific standard.

# Reviewer report: Reviewer response for version 1

**DOI:** 10.21956/mep.22250.r41379

**Published:** 2025-04-24

**Authors:** Nancy Dalgarno

**Affiliations:** 1 Office of Professional Development and Educational Scholarship, Queen's University, Kingston, Ontario, Canada

**Overall Comments**

 This is an interesting brief report that describes the importance of developing proficient teaching skills in residents and the need for a “Resident as Teacher curriculum”. However, the manuscript is confusing as there are times when I think that it is about evaluation of current residency training in medical education and other times about evaluating the teaching residents to teach initiative at UdeM. For example, in the first sentence of the discussion it states, “This project represents the first evaluation of current residency training in medical education at UdeM.”, this seems very different and much broader than evaluating the resident perspectives of teaching other residents. The manuscript needs to be reworked to ensure the purpose is clear throughout. Below are other suggestions that should be addressed if it is to be indexed.

**Abstract**

- Add UdeM after Université de Montréal the first time it is used in the introduction: Université de Montréal (UdeM)
- Add a period at the end of the last sentence in the introduction.
- Add a hyphen to “19-question…”
- Use past tense in the results section
- How were narrative comments analyzed. You state that descriptive statistics were used but not how the qualitative data was analyzed.

**Introduction**

- It needs to be explicitly clear early on that this is not the evaluation of current residency training in medical education, but rather an evaluation of a resident as teacher curriculum.
- Change CanMeds to CanMEDS
- Change “As of date,…” to “To date, …”
- In the second paragraph, relate the focus of the residency curriculum to the CanMEDS roles (e.g., medical expert)
- The sentence “Residents are often overwhelmed by competing responsibilities in patient care, research, and administration without forgetting that they are learners themselves.” doesn’t make sense. Is it suppose to be “…while forgetting that they…”?
- Spell “OR” in full the first time it is used.
- Make sure the purpose clearly states it’s about residents as teachers and not about evaluating the full training medical education program/curriculum. For example, the first sentence in the ‘goals’ section state “The goal of this project was to assess the interest of junior residents in medical education and the need to develop further training at our institution.”, medical education needs to be defined here to specifically state that it’s about residents as teachers.

**Methods**

- In the statement “Confidence in teaching skills evaluated in the elective course at UdeM was assessed by using a 5-point Likert scale, where 1 was least comfortable and 5 was most comfortable.”, you are measuring ‘comfort’ not confidence according to the scale. Please correct.
- Include the survey as an appendix.
- It appears that the survey was adequately reviewed and tested.
- More detail is needed about how the narrative comments were analyzed, rather than thematically (e.g., how many researchers analyzed them, was there inter-rater reliability).
- A reflexivity statement is needed.

**Results**

- **“**Supervision of their teaching (18 %), classes on education theory (14 %), online modules (12 %), and small group activities (11 %) also had interest.” is an awkward sentence. Please consider rewording.
- Avoid using ‘however’ at the beginning of a paragraph.
- In the sentence “Most residents are somewhat comfortable with most skills, especially as professional role models.” please identify what “most skills” were.
- Please state the number of themes and what they were in the first sentence (e.g., Three themes emerged from the narrative analysis: Obstacles to training, Appreciation of current curriculum, and Skills to improve.)
- Results should be written in past tense. Please correct. (e.g., change “One responder believed that it can be difficult…” to “One responder believed that it could have be difficult…”

**Discussion**

- See comments above re: first sentence.
- Suggest connecting it back to Can MEDS scholar role that you introduced in the introduction.

**Limitations and recommendations**

- Separate limitations and recommendations into 2 sections.
- Interesting and relevant recommendations.

**Conclusion**

- It’s great that teaching as part of a resident’s role is explicitly stated in the conclusion. This needs to be done throughout the manuscript; it is just one part of training within the medical education field.

**References**

- Add DOIs to each journal

Have any limitations of the research been acknowledged?

Yes

Is the study design appropriate and does the work have academic merit?

Yes

Is the work clearly and accurately presented and does it cite the current literature?

Partly

If applicable, is the statistical analysis and its interpretation appropriate?

Partly

Are all the source data underlying the results available to ensure full reproducibility?

No

Are the conclusions drawn adequately supported by the results?

Yes

Are sufficient details of methods and analysis provided to allow replication by others?

Yes

Reviewer Expertise:

Medical education, program evaluation

I confirm that I have read this submission and believe that I have an appropriate level of expertise to confirm that it is of an acceptable scientific standard, however I have significant reservations, as outlined above.

# Response

**Published:** 2025-09-29

**Authors:** Yi Zhen Jia

**Affiliations:** Medicine, Universite de Montreal, Montreal, Québec, Canada

Thank you very much for your review of this manuscript. **Abstract**

1. Grammar suggestions were changed.
2. Analysis of qualitative data has been clarified in the methods section.

**Introduction**

- Grammar suggestions were changed.
- Clarification that this is a RaT curriculum evaluation and not of the entire medical education curriculum was done.

**Methods**

- Grammar suggestions were changed.
- The survey is included in the repository.
- Clarification of narrative comments was done including number of researchers and variability.
- Reflexivity statement was added.

**Results**

- Grammar suggestions were changed.
- The number of themes found in the analysis and the skills that residents were comfortable with was clarified.

**Discussion**

- As above.

**Limitations and recommendations**

- Limitations and recommendations were separated.

**Conclusion**

- Teaching as a resident’s role was reinforced in the manuscript.

**Reference**

- The DOIs of each reference was clarified. 

# Reviewer report: Reviewer response for version 1

**DOI:** 10.21956/mep.22250.r41384

**Published:** 2025-04-16

**Authors:** Sacha Sharp

**Affiliations:** 1 Indiana University School of Medicine, Indianapolis, Indiana, USA

***Is the study design appropriate and does the work have academic merit?***

 Please provide more information in the study design section related to conceptual framework and why certain decisions were made pertaining to the survey development. 

***Are sufficient details of methods and analysis provided to allow replication by others?***

 This study would be hard to replicate with the limited information provided. 

***If applicable, is the statistical analysis and its interpretation appropriate?***

 Because I am a qualitative researcher by training, I cannot comment on the statistical analysis. That said, I would have liked to see more disaggregation of the data outside of just the resident specialties. This could have allowed for richer analysis. 

***Are the conclusions drawn adequately supported by the results?***

 The conclusions and recommendations are extremely limited and some conflict with the results. More discussion is needed for how to promote more resident teacher education when barriers are present. 

**In the Manuscript:**

***Introduction ***

- The first line is confusing, "up to half of medical students.." and the citations are dated. May you cite more recent literature on the topic of resident and medical student interactions?
- May you share more details about Can Meds scholar competency for the international audience who is unfamiliar?
- In paragraph two, please edit sentence - "Residents are often overwhelmed by competing responsibilities in patient care, research, and administration *without forgetting* that they are learners themselves" - for clarity. 
- Can you share more information that confirms the lack of teacher education at your institution is a problem? Have the medical students' or faculty highlighted challenges? 

***Methods***

- Was this survey validated with survey administrators? I see that residents reviewed it as well as some faculty, but what other validation process was conducted? Why was the survey sent to PGY1s - PGY3s if the idea was about junior residents and their teaching? Maybe I misunderstand what you mean by junior? Is there a conceptual or theoretical framework the group used to undergird the study? I am assuming resident as teacher could be a good framework, but more explanation is needed to help us understand how that framework is used to develop the study and analyze the results. 

***Results***

- I would encourage a stronger synthesis of the data. The information provided is not detailed enough. Instead of just listing what the results say, help us making meaning of the results in the context of your medical education system and the current literature on the topic. 

***Discussion***

- Share more about whether your findings confirm or deny literature about this topic. Dig deeper. How can the residency curriculum be adjusted to make space for teaching? What are other residents as teacher programs doing? What are the systemic issues at your institution that exacerbate this problem? What does varied and flexible courses mean? How do you shift the culture to one of teaching at an institution?

***Recommendations***

- These need to be explained. Some of these recommendations conflict with the findings and the literature. 

Have any limitations of the research been acknowledged?

Yes

Is the study design appropriate and does the work have academic merit?

Partly

Is the work clearly and accurately presented and does it cite the current literature?

Yes

If applicable, is the statistical analysis and its interpretation appropriate?

I cannot comment. A qualified statistician is required.

Are all the source data underlying the results available to ensure full reproducibility?

Yes

Are the conclusions drawn adequately supported by the results?

No

Are sufficient details of methods and analysis provided to allow replication by others?

No

Reviewer Expertise:

I serve as director of medical education innovation at my institution, I work directly with our resident as teacher (RasT) programs, and I have published on RasT programming. My areas of research pertain to medical education across the continuum, teaching in the clinical environment, and the experiences of underrepresented learners in medicine.

I confirm that I have read this submission and believe that I have an appropriate level of expertise to confirm that it is of an acceptable scientific standard, however I have significant reservations, as outlined above.

# Response

**Published:** 2025-09-29

**Authors:** Yi Zhen Jia

**Affiliations:** Medicine, Universite de Montreal, Montreal, Québec, Canada

Thank you very much for your review of this manuscript. Suggestions were incorporated in the manuscript. Study design and conceptual framework have been further explained. There was difficulty for further disaggregation of data due to the limited response rate. **Introduction**

- I have not found much of high-quality more recent literature on resident-medical student interactions. I have added one reference that I found.
- CanMEDS competency of scholar was further detailed.
- Request for participation in the elective for teaching as well as more opportunities was highlighted.

**Methods**

- The concept of junior residents in Canada was detailed.
- Study design and conceptual framework have been further stated and explained.

**Results**

- Synthesis of data was reworked. Meaning of the results were further detailed in the discussion.

**Discussion**

- Comparison with the US residency training and literature was done.
- RaT curriculum of other universities was provided.
- Systemic issues and challengers proper to our institution was included.
- Ideas for bigger changes in structures were shared.

**Recommendations**

- Some of the recommendations were reworked. We believe that they are in alignment with current literature and this study.
